# Optimization of physical quantities in the autoencoder latent space

**DOI:** 10.1038/s41598-022-13007-5

**Published:** 2022-05-30

**Authors:** S. M. Park, H. G. Yoon, D. B. Lee, J. W. Choi, H. Y. Kwon, C. Won

**Affiliations:** 1 Department of Physics, Kyung Hee University, Seoul, 02447 South Korea; 2 Center for Spintronics, Korea Institute of Science and Technology, Seoul, 02792 South Korea

**Keywords:** Ferromagnetism, Scientific data

## Abstract

We propose a strategy for optimizing physical quantities based on exploring in the latent space of a variational autoencoder (VAE). We train a VAE model using various spin configurations formed on a two-dimensional chiral magnetic system. Three optimization algorithms are used to explore the latent space of the trained VAE. The first algorithm, the single-code modification algorithm, is designed for improving the local energetic stability of spin configurations to generate physically plausible spin states. The other two algorithms, the genetic algorithm and the stochastic algorithm, aim to optimize the global physical quantities, such as topological index, magnetization, energy, and directional correlation. The advantage of our method is that various optimization algorithms can be applied in the latent space containing the abstracted representation constructed by the trained VAE model. Our method based on latent space exploration is utilized for efficient physical quantity optimization.

## Introduction

The optimization process is the search for the optimal solution among a large set of possible solutions under given constraints defining a system. It is a challenging problem in various research fields including science, and various numerical methods have been extensively utilized to solve optimization problems. In material science, optimal molecular structures have been successfully investigated based on density functional theory^1–3^. Simulated annealing (SA) is a conventional method to obtain an approximate global optimum^4–6^, and a genetic algorithm has also been used for designing molecules with desired properties^7,8^. Recently, the machine learning technique has also exhibited great potential in solving optimization problems. For example, unsupervised machine learning algorithms were used to find the ground state of a two-dimensional magnetic system^9,10^ and a generalized neural-network method was presented for constructing potential-energy surfaces based on density functional theory^11^.

The optimization methods can be combined with deep generative models, e.g., generative adversarial networks (GAN)^12^ or variational autoencoders (VAE)^13^, for various purposes. A deep generative model denotes deep neural networks trained to approximate complicated high-dimensional probability distributions using given samples^14^. Once a deep generative model is successfully trained, it can convert randomly sampled instances into real space data using the deep generative model. In previous studies^15,16^, evolutionary algorithms were adopted to explore the latent space of a trained generative model, where the latent space denotes a virtual space from which the random instances are located at to be converted into meaningful data. Since the generative model is constructed by differentiable neural networks, we can use gradient-based optimization methods^17^. Gomez-Bombarelli et al.^18^ applied a gradient-based method in the latent space of trained VAE to optimize molecules via their properties. Similarly, if we combine various optimization methods with deep generative models trained on physical data, several advantages are expected in solving various optimization problems in physics. Specifically, a well-trained deep generative model obeying the constraints of the system will enable to search for the solutions of optimization problems under those constraints.

In this study, we train a VAE on our dataset composed of various spin configurations formed on a two-dimensional magnetic system. We explore the VAE latent space to obtain optimal physical states. To search for the optimal solution by exploring the latent space of the trained VAE network, three optimization algorithms are implemented: the single-code modification algorithm, genetic algorithm, and stochastic algorithm. Using these algorithms, we obtain either the local or global optimal states that are representative of the system but not included in the training dataset and propose that our methods have the potential to be applied to various optimization problems in scientific research fields.

## Strategy

### Dataset

The dataset should be chosen to contain rich features within a large variety of possible spin states, so that our VAE network can properly learn to abstract input spin configurations to latent codes and decode the abstracted information to spin configurations. In this study, the labyrinth spin configurations^19^ which have various maze patterns constructed by magnetic spiral structures are selected as our dataset.

**Figure 1:**
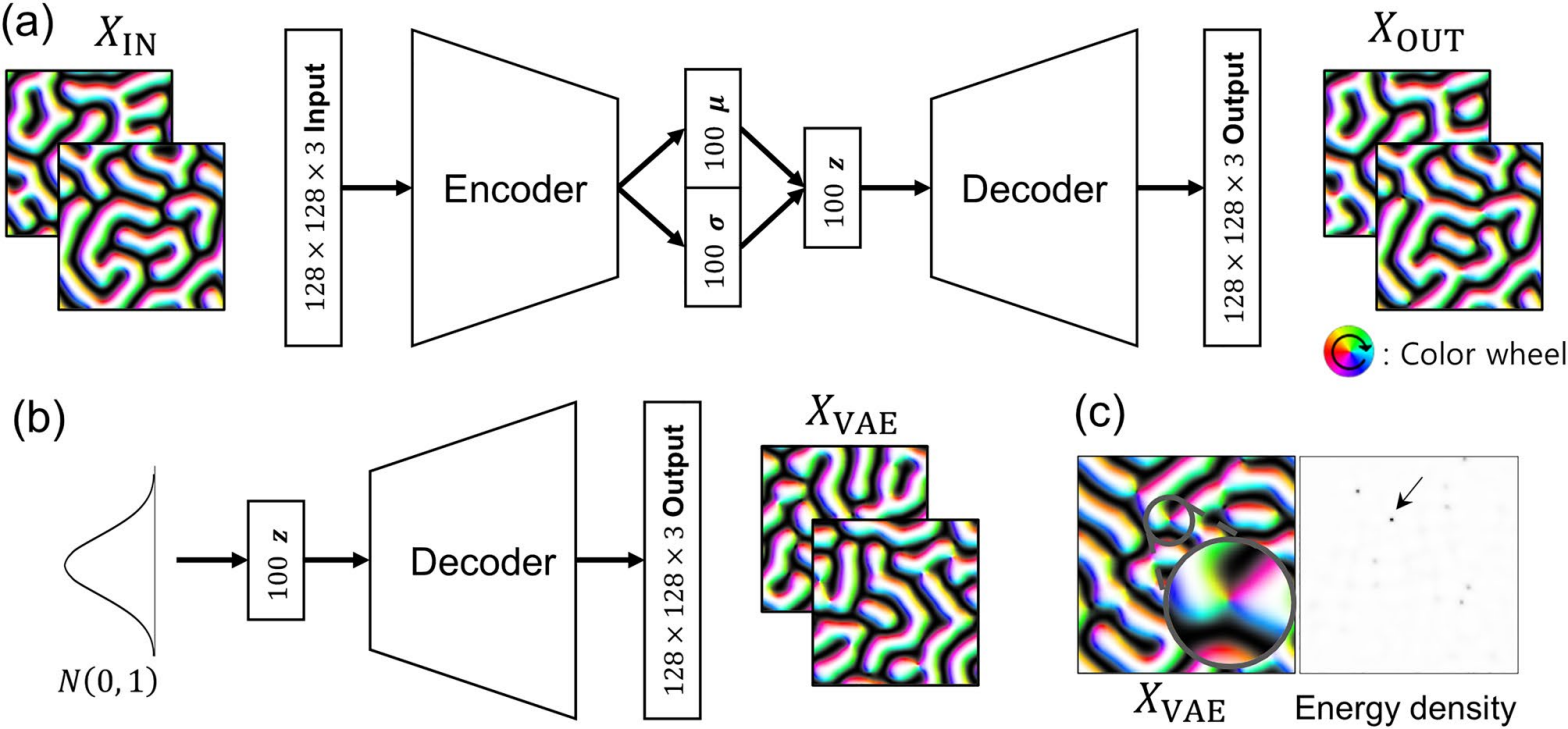
(**a**) Schematic diagram of the variational autoencoder and examples of ${X}_{\mathrm{IN}}$ and ${X}_{\mathrm{OUT}}$. The color wheel and black/white contrast of the spin configuration images indicate the in-plane and out-of-plane magnetization directions, respectively. (**b**) A schematic illustration of the generation process of ${X}_{\mathrm{VAE}}$. (**c**) A representative ${X}_{\mathrm{VAE}}$ image and its local energy density. The inset in the spin configuration image of (**c**) shows a magnified view of a nodal point. The arrow in the energy density image indicates the location of high energy density and it corresponds the position of the nodal point.

To generate the labyrinth spin configurations, we implement the two-dimensional magnetic system using a square lattice model composed of $128\times 128$ grid sites and consider a simple Hamiltonian model, $\mathcal{H}=-J{\sum }_{\langle i,j\rangle }{\overrightarrow{S}}_{i}\cdot {\overrightarrow{S}}_{j}-{\sum }_{\langle i,j\rangle }{\overrightarrow{D}}_{ij}\cdot \left({\overrightarrow{S}}_{i}\times {\overrightarrow{S}}_{j}\right)$, where $J$ is the exchange interaction parameter, ${\overrightarrow{D}}_{ij}$ is the Dzyaloshinskii-Moriya (DM) interaction vector^20,21^, and ${\overrightarrow{S}}_{i}$ is a Heisenberg spin on the $i$-th grid site. The exchange and DM interaction parameters are fixed to $J=1$ and $\left|{\overrightarrow{D}}_{ij}\right|=0.3$. The system was carefully studied, so that we can investigate and interpret the results from our algorithm by relying on those of previous studies^20–24^. The dataset is generated by an SA process, which has been used to simulate a temperature annealing process of magnetic systems in several previous studies^5,19,25^. Since the detailed magnetic textures are determined by the spontaneous symmetry breaking process, they can have countless different metastable states in a fixed condition. Using this process, we generate a training dataset consisting of 30,000 labyrinth spin configurations, which is called ${X}_{\mathrm{IN}}$ in this study, and some samples are shown in Fig. 1a.

Since our purpose is to find optimization solutions, it is important that the training data are sampled from entire data distribution. The SA process is performed by heat bath algorithm in Monte-Carlo simulations^26^, a carefully studied method. This method can generate a variety of samples due to the contribution of random numbers of it.

### Training VAE and generating new data

To extract the appropriate features implied in the labyrinth spin configurations, we construct the VAE network as shown in Fig. 1a. Specifically, the encoder of the VAE compresses ${X}_{\mathrm{IN}}$ into two groups composed of $N$ numbers each ($N=100$ in this study). These are used as the mean (${\varvec{\mu}}$) and standard deviation (${\varvec{\sigma}}$) values to construct $N$ normal distributions, and one number is sampled from each of the normal distributions ($N$ numbers are sampled in total). In general, the set of sampled numbers, ${\varvec{z}}$, is called the latent code; ${\varvec{z}}$ is considered an $N$-dimensional vector represented in the latent space. Through the decoder, ${\varvec{z}}$ is decoded into an output spin configuration, ${X}_{\mathrm{OUT}}$.

We train the VAE network using our spin configuration dataset. In other words, the network parameters are modified to minimize a loss function, ${L}_{\mathrm{VAE}}$, shown in Eq. (1), where ${L}_{\mathrm{RC}}$ is the reconstruction loss term, ${L}_{\mathrm{KL}}$ is the Kullback–Leibler (KL) loss term, and β is the coefficient of ${L}_{\mathrm{KL}}$^27^. The two loss terms, ${L}_{\mathrm{RC}}$ and ${L}_{\mathrm{KL}}$, are expressed in Eq. (2), where ${S}_{\mathrm{IN},i,\alpha }$ and ${S}_{\mathrm{OUT},i,\alpha }$ indicate the $\alpha$ component of spins at the $i$-th grid site of ${X}_{\mathrm{IN}}$ and ${X}_{\mathrm{OUT}}$, respectively. ${\mu }_{n}$ and ${\sigma }_{n}$ are the $n$-th components of ${\varvec{\mu}}$ and ${\varvec{\sigma}}$, respectively.

1 $${L}_{\mathrm{VAE}}={L}_{\mathrm{RC}}+\beta {L}_{\mathrm{KL}},$$

2 $$\begin{gathered} L_{{{\text{RC}}}} = \left( {S_{{{\text{IN}},i,\alpha }} - S_{{{\text{OUT}},i,\alpha }} } \right)^{2}_{{i,\alpha \left( { = x,y,z} \right)}} \hfill \\ L_{{{\text{KL}}}} = \frac{1}{2}\mathop \sum \limits_{n = 1}^{N} \left( {\sigma_{n}^{2} + \mu_{n}^{2} - \ln \left( {\sigma_{n}^{2} } \right) - 1} \right). \hfill \\ \end{gathered}$$

To minimize ${L}_{\mathrm{RC}}$, the VAE model produces ${X}_{\mathrm{OUT}}$, which is similar to ${X}_{\mathrm{IN}}$. ${L}_{\mathrm{KL}}$ measures how a given probability distribution is different from a reference probability distribution. In this study, the purpose of ${L}_{\mathrm{KL}}$ is to make the probability distribution of ${\varvec{z}}$ equal to the standard normal distribution; as ${L}_{\mathrm{KL}}$ decreases, the values of ${\sigma }_{n}$ and ${\mu }_{n}$ approach 1 and 0, respectively. $\beta$ is chosen to be 0.001, as described in the Methods section.

After the training process, we can generate various spin configurations by decoding the latent codes that are sampled from the standard normal distribution. The generation process is illustrated schematically in Fig. 1b, and the spin configurations generated using this process are called ${X}_{\mathrm{VAE}}$s in this study. The ${X}_{\mathrm{VAE}}$s have similar characteristics to the spin configurations in our training dataset. This indicates that the VAE network is well-trained to learn the magnetic characteristics implied in the training dataset, such as the spin profiles, length scale, and chirality.

There are several nodal points in the ${X}_{\mathrm{VAE}}$s, which are visually unfeasible and not found in the training dataset. We can also quantitatively evaluate the feasibility by calculating the energy of ${X}_{\mathrm{VAE}}$ since the energy is the generating rule of the training dataset. The energy density of nodal points is higher than that of other plausible pixels, as indicated by the energy peak points in Fig. 1c. Unstable points are inevitable from the direct interpretation of the sampled ${\varvec{z}}$, since the latent space is continuous while the stable structures are topologically separated. This problem of directly applying VAE to a topologically discrete structure was reported in a previous study^10^.

### Latent space exploration algorithms

**Figure 2:**
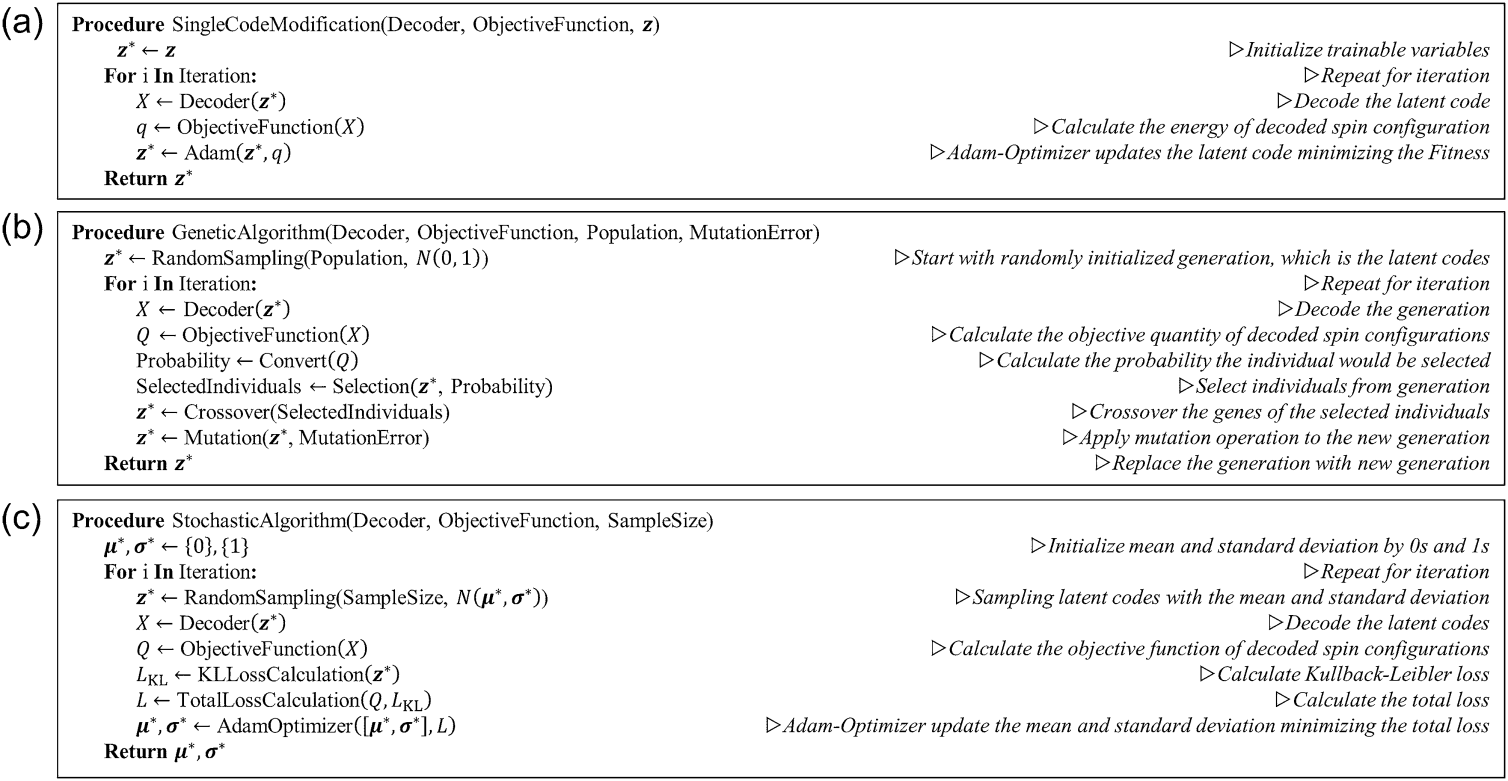
Pseudocode descriptions of the (**a**) single-code modification algorithm, (**b**) genetic algorithm, and (**c**) stochastic algorithm.

To generate physically plausible data or to obtain the optimized structure using the trained VAE decoder, we attempt three optimization algorithms exploring the latent space. The first is a simple optimization algorithm, which is called the single-code modification algorithm in this study. The algorithm pseudocode is shown in Fig. 2a. We prepare a latent code, ${{\varvec{z}}}^{\boldsymbol{*}}$, composed of $N$ trainable variables and initialize it with ${{\varvec{z}}}^{\boldsymbol{*}}={\varvec{z}}$, where ${\varvec{z}}$ is randomly sampled numbers from the standard normal distribution. ${{\varvec{z}}}^{\boldsymbol{*}}$ is decoded into a spin configuration using the trained decoder and evaluated with its energy. The Adam optimizer^28^ modifies ${{\varvec{z}}}^{\boldsymbol{*}}$ to minimize the spin configuration energy. This process is repeated until the energy stabilizes. The main idea of the single-code modification algorithm is to search the latent space to find a better solution near the position indicated by the selected initial latent code. Thus, the states generated by the single-code modification algorithm are locally optimized states in the latent space, and they are not uniquely determined.

If we want to find a state representing a global optimum state, the whole latent space should be searched. To utilize the idea for the general optimization problem, we apply a genetic algorithm instead of the single-code modification algorithm. The genetic algorithm is one of the conventional optimization methods imitating biological evolution. In the algorithm, there is a population composed of many individuals, and the population evolves to optimize an objective goal. Every individual consists of their genes. Individuals are evaluated with the objective function and selected to pass on their genes to the next generation with a probability calculated from the evaluation. Crossover and mutation operations are performed to combine the genes of the selected individuals and to maintain the genetic diversity, respectively. The pseudocode of the genetic algorithm used in this study is shown in Fig. 2b. We consider ${{\varvec{z}}}^{\boldsymbol{*}}$ as an individual and every number $\left\{{z}_{i}^{*}\right\}$ of ${{\varvec{z}}}^{\boldsymbol{*}}$ is considered its gene. Our population is composed of $100$ individuals, ${{\varvec{z}}}^{\boldsymbol{*}}$ s, which are randomly initialized. In every iteration of the genetic algorithm process, ${{\varvec{z}}}^{\boldsymbol{*}}$ s are decoded and evaluated with an objective function. In this study, we use several quantities that can be calculated from a spin configuration as the objective function, which is discussed in the Results section.

The third algorithm, which is the stochastic algorithm, is illustrated in Fig. 2c. The algorithm is similar to the single-code modification algorithm but is designed for global optimization. In this case, we update ${{\varvec{\mu}}}^{\boldsymbol{*}}$ and ${{\varvec{\sigma}}}^{\boldsymbol{*}}$ instead of ${{\varvec{z}}}^{\boldsymbol{*}}$, which denotes the mean and standard deviation values of normal distributions. ${{\varvec{\mu}}}^{\boldsymbol{*}}$ and ${{\varvec{\sigma}}}^{\boldsymbol{*}}$ are each composed of $N$ trainable variables, and they are initialized with $0$ s and $1$ s, respectively. In every training iteration, hundreds of ${{\varvec{z}}}^{\boldsymbol{*}}$ are sampled from $N$ normal distributions constructed using ${{\varvec{\mu}}}^{\boldsymbol{*}}$ and ${{\varvec{\sigma}}}^{\boldsymbol{*}}$ values, and the ${{\varvec{z}}}^{\boldsymbol{*}}$ s are decoded into output spin configurations using the trained decoder. They are evaluated with a given objective quantity, $Q$, similar to the case of the genetic algorithm. The Adam optimizer updates the ${{\varvec{\mu}}}^{\boldsymbol{*}}$ and ${{\varvec{\sigma}}}^{\boldsymbol{*}}$ to minimize the function, $L=\langle Q\rangle +\gamma {L}_{\mathrm{KL}}^{*}$, where ${L}_{\mathrm{KL}}^{*}$ is a term similar to the KL loss term shown in Eq. (2) except that it is calculated using ${{\varvec{\mu}}}^{\boldsymbol{*}}$ and ${{\varvec{\sigma}}}^{\boldsymbol{*}}$, and $\gamma$ is the coefficient of ${L}_{\mathrm{KL}}^{*}$. ${L}_{\mathrm{KL}}^{*}$ is adopted to control the diversity of the ${{\varvec{z}}}^{\boldsymbol{*}}$ s, and it is expected that this diversity can prevent the distribution from being trapped in local extremum points in the latent space. As the iteration progresses, we gradually decrease $\gamma$ so that the distribution slowly converges to the optimal solution in the latent space.

## Results

### Single-code modification algorithm

**Figure 3:**
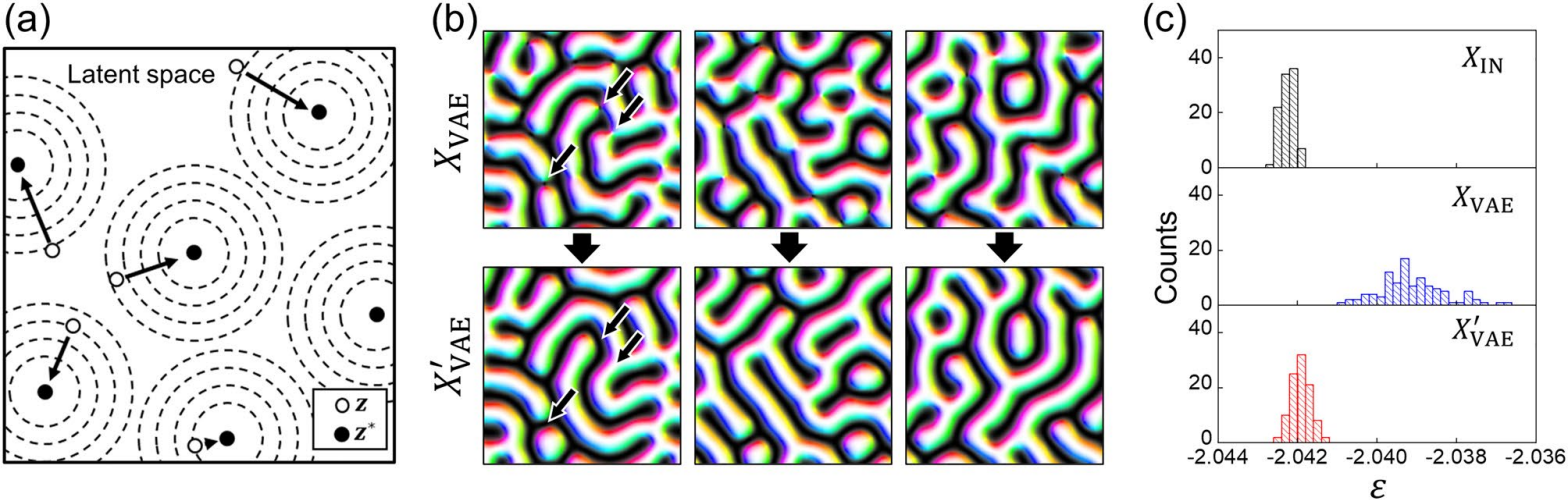
The results of the single-code modification algorithm. (**a**) A schematic illustration of the algorithm. The empty circles, filled circles, and dashed lines illustrate the initial latent code ${\varvec{z}}$, modified latent code ${{\varvec{z}}}^{\boldsymbol{*}}$, and contour line of the energy, respectively. (**b**) Examples of the generated spin configuration ${X}_{\mathrm{VAE}}$ and the modified spin configuration ${X}_{\mathrm{VAE}}^{^{\prime}}$. The arrows on the leftmost ${X}_{\mathrm{VAE}}$ image indicate nodal points, and the arrows on the leftmost ${X}_{\mathrm{VAE}}^{^{\prime}}$ image indicate the same regions, showing that the nodal points are removed. (**c**) The $\varepsilon$ distributions of ${X}_{\mathrm{IN}}$s, ${X}_{\mathrm{VAE}}$s, and ${X}_{\mathrm{VAE}}^{^{\prime}}$s. The distribution was gathered from 100 spin configurations of each case.

The purpose of this algorithm is to generate new structures that have similar characteristics to those in the training data, and Fig. 3a shows a schematic illustration of the algorithm. As described earlier, ${X}_{\mathrm{VAE}}$s decoded from ${\varvec{z}}$s have many nodal points that are not included in the training data, which means that direct sampling from the standard normal distribution is not enough to generate physically plausible states. Hence, we perform the single-code modification algorithm to search the better positions near ${\varvec{z}}$s, which can be decoded into energetically plausible states. Figure 3b shows some examples of ${X}_{\mathrm{VAE}}$ and ${X}_{\mathrm{VAE}}^{^{\prime}}$, where ${X}_{\mathrm{VAE}}^{^{\prime}}$ represents the modified spin configuration after applying this algorithm. As indicated by the black arrows, the spin structures around the nodal points are revised to stable structures; the nodal points are removed. Figure 3c shows the distributions of energy density, $\varepsilon$, of ${X}_{\mathrm{IN}}$, ${X}_{\mathrm{VAE}}$, and ${X}_{\mathrm{VAE}}^{^{\prime}}$. The distribution of ${X}_{\mathrm{VAE}}^{^{\prime}}$ is similar to that of ${X}_{\mathrm{IN}}$, but there is a clear difference for the case of ${X}_{\mathrm{VAE}}$. This also indicates that we can generate spin configurations analogous to ${X}_{\mathrm{IN}}$ not only structurally but also physically and energetically using the single-code modification algorithm.

Of course, it is possible to incorporate the energy of ${X}_{\mathrm{VAE}}$s in the loss function of VAE so that ${X}_{\mathrm{VAE}}$s are forced to be energetically stable. A previous study^10^ shows that this approach is available at generating feasible and energetically stable samples in a magnetic system.

### Genetic algorithm

Genetic algorithms are known as efficient optimization methods, but their effectiveness is strongly dependent on how we define the individuals and genes in a given system. In this study, a latent code, which is the abstracted information of a spin configuration, is considered an individual, and the components of the latent code, which characterize the properties of the spin configuration, are considered the genes. By doing so, we perform genetic algorithm in a compressed space of ${\mathbb{R}}^{100}$ rather than the original space ${\mathbb{R}}^{128\times 128\times 3}$, reducing computational cost.

**Figure 4:**
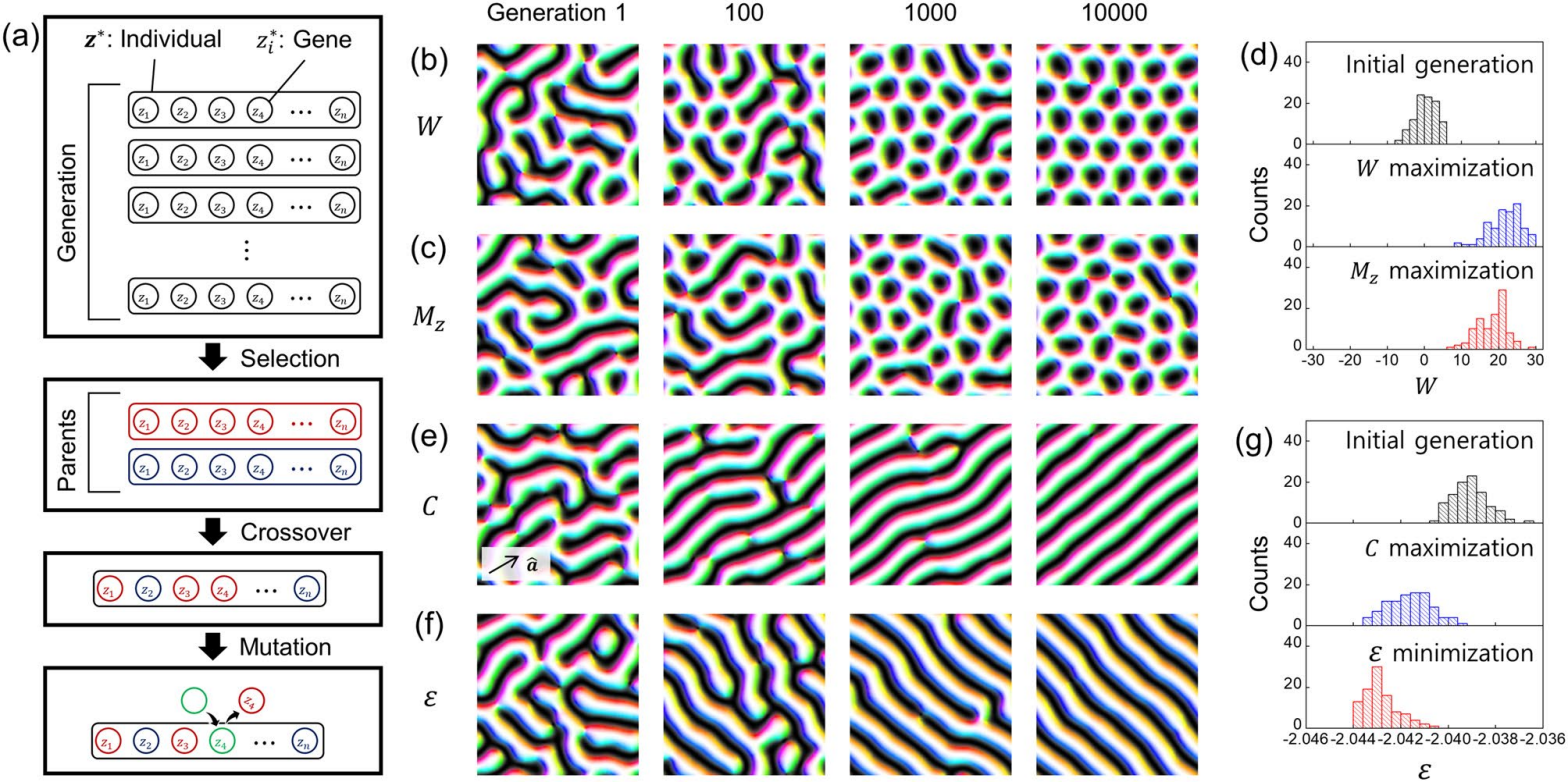
Results of the genetic algorithm. (**a**) A schematic illustration of the algorithm. The circles and rounded squares indicate the genes (components ${z}_{i}^{*}$) and individuals (latent code ${{\varvec{z}}}^{\boldsymbol{*}}$), respectively. The blue/red colors indicate each selected parent, and the green circle indicates the mutated gene. (**b,c,e,f**) The best individuals in each generation optimizing (**b**)$W$, (**c**)${M}_{z}$, (**e**)$C$, and (**f**)$\varepsilon$, respectively. The arrow in (**e**) indicates the direction of ${\varvec{a}}$. (**d**) $W$ values of spin configurations evolved with $W$ maximization and ${M}_{z}$ maximization, (**g**) $\varepsilon$ values of spin configurations evolved with $C$ maximization and $\varepsilon$ minimization.

A schematic explanation of this algorithm is shown in Fig. 4a. We start from 100 individuals initialized with random numbers sampled from the standard normal distribution. To determine the probability that an individual will be selected, the spin configuration decoded from the individual is evaluated by a certain objective quantity. The mutated gene, an instance of an offspring, is sampled from the Gaussian distribution, so that the offspring keeps in the learned distribution by the VAE, following the general features of training spin configurations.

We experiment with two structural quantities and two physical quantities for the evaluation. The structural quantities are the topological index, $W=\frac{1}{4\pi }\int {\varvec{M}}\cdot \left(\frac{\partial {\varvec{M}}}{\partial x}\times \frac{\partial {\varvec{M}}}{\partial y}\right)dxdy$, and the spin correlation along a certain direction pointed by a unit vector ${\varvec{a}}$, $C=\langle {\varvec{S}}\left({\varvec{r}}\right)\cdot {\varvec{S}}\left({\varvec{r}}+{\varvec{a}}\right)\rangle$. The physical quantities are the energy density, $\varepsilon$, indicated by the Hamiltonian of the system and the out-of-plane magnetization, ${M}_{z}$. Figure 4b,c,e,f show the best individuals of each generation evolved by the genetic algorithm to maximize $W$, maximize ${M}_{z}$, maximize $C$, and minimize $\varepsilon$, respectively.

As shown in Fig. 4b, the spin configuration is changed from the labyrinth structure to the skyrmion structure^29^ by maximizing $W$. The spin configuration of the best individual in the last generation almost forms a regular lattice of skyrmions, and it is a noticeable result because the skyrmion lattice structure is not included in the training set of ${X}_{\mathrm{IN}}$s; the skyrmion lattice structure is one of the possible metastable states of the system, but it is statistically impossible to generate it using an SA process due to the existence of numerous metastable states of the system. This means that the VAE model has learned the general features of the system, such as the chirality, the width of magnetic domains, and sinusoidal profiles of local spin structures, and a set of optimal features to maximize $W$ is properly searched through the genetic algorithm in the latent space of the trained VAE.

Interestingly, a similar skyrmion structure is obtained by maximizing ${M}_{z}$, as shown in Fig. 4c. In numerous studies^24,30–32^, it is commonly observed that the labyrinth structure becomes a magnetic skyrmion configuration when an external field is applied to the out-of-plane direction of the two-dimensional magnetic system. Since the out-of-plane external field usually increases ${M}_{z}$ to reduce the Zeeman energy, it is reasonable to show similar structures as the result of maximizing ${M}_{z}$ and $W$ in our methods. Figure 4d shows the distributions of $W$ values calculated from the spin configurations evolved by the genetic algorithm to maximize $W$ and ${M}_{z}$ for each. We find that the $W$ values from both optimizations are increased compared with those from the initial generation.

It is also noticeable that the optimization is performed within the bounds of the system, producing feasible and realizable results as commonly observed in experimental research^24,31^. Since $W$ counts the number of skyrmion structures in a spin configuration, breaking the spin structures into smaller structures is one way to maximize $W$, yet this is not compatible with the Hamiltonian of the system as well as not feasible in experimental observations. ${M}_{z}$ can also be maximized by generating a single out-of-plane domain along the $z$ direction, but this is also incompatible with the Hamiltonian. Although these are very intuitive methods for maximizing $W$ and ${M}_{z}$, our method takes more suitable approaches to the Hamiltonian of the system during the optimization processes, as shown in Fig. 4b–d. This shows that our method is a physically plausible optimization method working within the constraints of a system.

By maximizing $C$, we obtain a well-aligned stripe pattern as shown in Fig. 4e; the stripes are aligned along the direction of ${\varvec{a}}$. A well-aligned stripe pattern is also generated by minimizing $\varepsilon$, as shown in Fig. 4f. Similar to the skyrmion cases discussed above, it is also noticeable that the results of both optimization processes, maximizing $C$ and minimizing $\varepsilon$, are the well-aligned stripe patterns because there are no such spin configurations in our training dataset. The stripe pattern is the ground state of this system^19^. Nevertheless, generating the ground state through an SA process is significantly difficult due to the existence of countless labyrinth patterns that are the metastable states of the system. The $\varepsilon$ of both results, obtained by maximizing $C$ and minimizing $\varepsilon$, decrease, as shown in Fig. 4g. Considering our method can search out the well-aligned stripe patterns that are not given in the training process, we believe that our method can be utilized as a novel approach to finding uncharted ground states of various systems. As discussed in “Training VAE and generating new data” section, energy is a proper indicator to evaluate the feasibility of spin configurations. Therefore, it can be interpreted that we ranked the offspring based on the feasibility while optimizing the energy with the genetic algorithm.

Our results show that VAEs are advantageous for applying genetic algorithms to optimization problems. Results of the algorithm are optimal solutions following the general rules of the system. Various metrics, such as discriminator networks of GANs or pre-trained classifiers, can be adapted for those purposes.

### Stochastic algorithm

**Figure 5:**
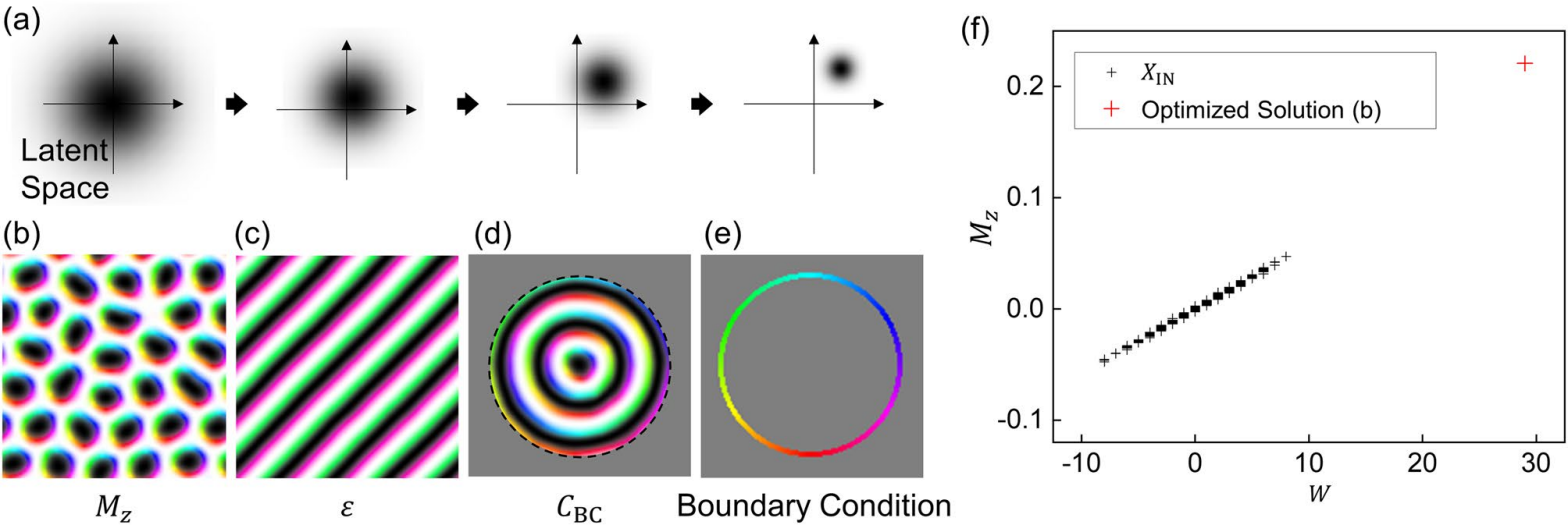
The results of the stochastic algorithm. (**a**) A schematic illustration of how the algorithm progresses. The blurs indicate the Gaussian distributions constructed with ${{\varvec{\mu}}}^{\boldsymbol{*}}$ (mean) and ${{\varvec{\sigma}}}^{\boldsymbol{*}}$ (standard deviation) in this algorithm. (**b–d**) Spin configuration obtained by (**b**) maximizing ${M}_{z}$, (**c**) minimizing $\varepsilon$, and (**d**) maximizing ${C}_{\mathrm{BC}}$. The boundary condition is given along the dashed line. (**e**) Illustration of the boundary condition ${{\varvec{S}}}_{\mathrm{BC}}$. (**f**) $W$ and ${M}_{z}$ of 1000 ${X}_{\mathrm{IN}}$s and the optimized solution shown in (**b**).

In this algorithm, there is a Gaussian distribution from which the latent codes,${{\varvec{z}}}^{\boldsymbol{*}}$, are sampled, and the mean, ${{\varvec{\mu}}}^{\boldsymbol{*}}$, and standard deviation, ${{\varvec{\sigma}}}^{\boldsymbol{*}}$, of the distribution are updated by the Adam optimizer to optimize an objective quantity. For each optimization iteration, we sampled 100 ${{\varvec{z}}}^{\boldsymbol{*}}$s from the distribution and decoded them to calculate an objective quantity similar to that of the genetic algorithm. As the algorithm progresses, the distribution gradually converges to a location in the latent space of the trained VAE, as schematically shown in Fig. 5a. We repeat this algorithm until the distribution converges sufficiently to a location and found that the latent codes sampled around the location can be decoded as the optimal solutions to given optimization problems.

Consequently, the skyrmion structures and perfectly aligned stripe pattern are obtained using this algorithm for the ${M}_{z}$ maximization and $\varepsilon$ minimization objectives, respectively, as shown in Fig. 5b,c. Compared with the results from the genetic algorithm, we find that the spin configurations obtained using this stochastic algorithm are more realistic in the visual inspection; in Fig. 5b,c, there are no nodal points and wiggles which are shown in the images for the last generations in Fig. 4b,c,e,f. Quantitatively, the maximum energy density of Fig. 5b is approximately $-1.88$, which is much lower than the energy peak ($\sim -0.11)$ in Fig. 1c. Note that we do not give any bias for reality or energetic stability in the optimization process.

Since VAEs model the given data, it is known that interpolation using VAEs works successfully providing reasonable results^33^. Our optimization results can also be understood as combinations of training data. For example, the skyrmion structures (see Figs. 4b,c, 5b) might be considered combinations of many training data that occasionally contain a few skyrmions. Nevertheless, as discussed in “Genetic algorithm” section, the skyrmion structure is outside the given data, not only in a visual inspection but also in physical quantities. Figure 5f shows the ${M}_{z}$ and $W$ of spin configurations. We found that the relation between ${M}_{z}$ and $W$ of ${X}_{\mathrm{IN}}$ is maintained in our optimized solution (b), even though it is an extreme situation. We confirm that it is possible to obtain desired optimal solutions using the stochastic algorithm, keeping the plausibility with the help of the VAE trained on samples.

We investigate whether this algorithm can be applied not only to a system with periodic boundary conditions but also to a geometrically confined system with a specific boundary shape. For this purpose, the objective quantity is set to be the correlation between a decoded spin configuration and the specific boundary condition map shown in Fig. 5d, ${C}_{\mathrm{BC}}=\langle {{\varvec{S}}}_{\mathrm{BC}}\left({\varvec{r}}\right)\cdot {\varvec{S}}\left({\varvec{r}}\right)\rangle$, where ${{\varvec{S}}}_{\mathrm{BC}}$ indicates the boundary condition. The boundary condition is illustrated in Fig. 5e. We perform an optimization process using this stochastic algorithm to maximize ${C}_{\mathrm{BC}}$. Since the spins in the boundary condition map are located only at the round narrow strip, maximizing ${C}_{\mathrm{BC}}$ presents geometrical confinement on the decoded spin configurations as shown in Fig. 5d. We confirmed that the spin configuration is in an energetically stable state, which means that this stochastic algorithm is applicable to find physically plausible states in various systems regardless of boundary conditions.

The idea of exploring the latent space of a VAE for generating optimized samples of a distribution given cost functions is innovative and promising as our results demonstrate. Our methods, single-code modification, genetic, and stochastic algorithms, are generally applicable if you can train deep generative models that provide compressed representations of the real world. Given the complexity of the distribution of the problem, the number of instances may increase substantially, which needs to be considered. Also, it must be noted that the success of optimization with trained deep generative models can be applications dependent. For image generation and optimization, the success might be more expected because there has been much research and development. For other systems, it can be challenging to train reliable VAEs and obtain optimal solutions. If the trained deep generative model does not contain the desired optimal solution, to find the solution in the latent space is hardly expected. It is widely known that prescribed models such as VAEs have better data coverage than implicit models such as GANs^34^. We recommend choosing and training the deep generative model considering its performance in terms of data coverage.

## Conclusion

In this study, we trained generative model, the variational autoencoder (VAE), to extract the features of a two-dimensional magnetic system. We implemented three algorithms, the single-code modification algorithm, genetic algorithm, and stochastic algorithm, to solve various optimizing problems by exploring the latent space of the trained VAE. The single-code modification algorithm obtains a metastable spin configuration by locally optimizing the energy minimization problem. The genetic algorithm and stochastic algorithm are applied to various global optimization problems, such as minimizing energy and maximizing topological numbers. We confirmed that, even though the optimal solutions are not given explicitly in the VAE model training process, the optimization algorithms successfully determine the optimal solutions in the latent space regardless of the boundary conditions of the system. We suggest that our method based on latent space exploration can be applied to various other systems to optimize their properties.

## Methods

### The network structure of VAE

**Figure 6:**
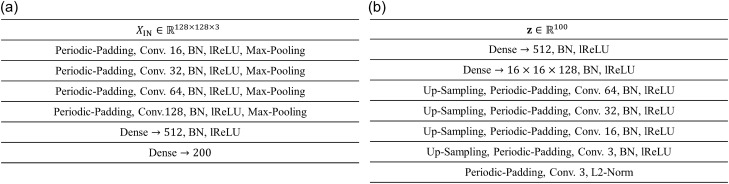
Detailed architectures of (**a**) the encoder and (**b**) decoder. The operations in a single block are written via its procedure. BN and lReLU represent batch normalization and leaky-ReLU activation layers, respectively.

The encoder and decoder of the VAE consist of fully connected neural networks (FCNNs) and convolutional neural networks (CNNs). Figure 6 shows the details of our architectures. The encoder is composed of four CNN layers with 16, 32, 64, and 128 filters and two FCNN layers with 512 and 200 neurons. After all CNN layers of the encoder, max-pooling layers with a $2\times 2$ pooling size are adopted. The decoder is composed of two FCNN layers with 512 and 8,192 neurons, the reshape layer, CNN layers with 64, 32, 16, 3, 3 kernels, and the L2-normalization layer. Upsampling layers with $2\times 2$ upsampling sizes are applied before all CNN layers of the decoder, except the last CNN layer. Batch normalization layers and leaky-ReLU activation layers are applied immediately after all FCNN and CNN layers, in both the encoder and the decoder, except for the last layer of each. Since the dataset is generated under the periodic boundary condition, periodic padding is applied immediately before all CNN layers.

### Detailed VAE training process

We used 30,000 spin configurations as a training dataset of VAE and used 5000 spin configurations as a valid dataset during training. The VAE is fed a batch of data at a time, where the batch consists of 100 spin configurations. The Adam optimizer with the learning rate ${10}^{-3}$ modifies the network parameters to minimize ${L}_{\mathrm{VAE}}$ every time the batch goes through the VAE. The ${\beta }_{1}$ and ${\beta }_{2}$ of the Adam optimizer are fixed at $0.9$ and $0.999$, respectively. The training progresses for 100 epochs.

To select the appropriate coefficients of ${L}_{\mathrm{KL}}$, $\beta$, we trained four VAE models with $\beta$ values of $0.1$, $0.01$, $0.001$, and $0.0001$. VAEs with $\beta$ larger than $0.001$ are excluded because of the posterior collapse problem^35^. We need a sufficiently compressed latent space so that the standard normal distribution represents the whole latent space. Thus, we chose the VAE of $\beta$ at $0.001$, which represents a latent code closer to the standard normal distribution.

### Single-code modification algorithm

The Adam optimizer with the learning rate ${10}^{-2}$ is used for the single-code modification. Other hyperparameters are identical to the Adam optimizer used for VAE training. The single-code modification algorithm is repeated for 10,000 iterations.

### Genetic algorithm

The genetic algorithm starts with 100 latent codes sampled from a standard normal distribution. Roulette wheel selection, uniform crossover, and random reset mutation operations are sequentially applied to construct a single new individual. The roulette wheel selection is applied to choose two parents^36^. We give the selection pressure differently depending on the objective quantity, as the selection pressure is a hyperparameter of the genetic algorithm. The selection pressure is given by 8 to optimize $W$ and ${M}_{z}$, and by 5 to optimize $C$ and $\varepsilon$. Uniform crossover is applied, which means that each gene ${z}_{i}^{*}$ is selected randomly from one of the corresponding genes of the parents^37^. Random reset mutation is applied so that each gene has a probability of being replaced by a new number generated from the standard normal distribution^38^. The mutation error, the probability that a gene will be mutated, is chosen by $0.01$. The process is repeated for 10,000 iterations.

### Stochastic algorithm

An Adam optimizer with the same hyperparameters as used in the single-code modification algorithm is used for the stochastic algorithm. $\gamma$ starts at 1 and is gradually decreased to a tenth after every 10,000 iterations. The process continues until ${\sigma }^{*}$ approaches zero so that the sampled latent codes are all the same.

## Data Availability

The dataset used in this work is available at https://data.mendeley.com/datasets/4833bhfjv3/1.
